# Supplementary material for: Subpathway Analysis of Transcriptome Profiles Reveals New Molecular Mechanisms of Acquired Chemotherapy Resistance in Breast Cancer
Source: Cancers (Basel). 2022 Oct 5;14(19):4878. doi: 10.3390/cancers14194878 (PMC9563670; doi:10.3390/cancers14194878)
Supplement: Supplementary file 1 [file cancers-14-04878-s001.zip › cancers-1808707-supplementary.pdf]

cancers MDPI

Table S1. Selected 65 Pathways.

Pathway ID	Pathway Name
hsa00010	Glycolysis/Gluconeogenesis
hsa00020	Citrate cycle (TCA cycle)
hsa00350	Tyrosine metabolism
hsa00360	Phenylalanine metabolism
hsa00564	Glycerophospholipid metabolism
hsa00565	Ether lipid metabolism
hsa00982	Drug metabolism
hsa04010	MAPK signaling pathway
hsa04012	ErbB signaling pathway
hsa04014	Ras signaling pathway
hsa04015	Rap1 signaling pathway
hsa04020	Calcium signaling pathway
hsa04022	cGMP-PKG signaling pathway
hsa04024	cAMP signaling pathway
hsa04060	Cytokine-cytokine receptor interaction
hsa04062	Chemokine signaling pathway
hsa04064	NF-kappa B signaling pathway
hsa04066	HIF-1 signaling pathway
hsa04068	FoxO signaling pathway
hsa04070	Phosphatidylinositol signaling system
hsa04071	Sphingolipid signaling pathway
hsa04072	Phospholipase D signaling pathway
hsa04080	Neuroactive ligand-receptor interaction
hsa04110	Cell cycle
hsa04114	Oocyte meiosis
hsa04115	p53 signaling pathway
hsa04122	Sulfur relay system
hsa04130	SNARE interactions in vesicular transport
hsa04141	Protein processing in endoplasmic reticulum
hsa04144	Endocytosis
hsa04145	Phagosome
hsa04146	Peroxisome
hsa04150	mTOR signaling pathway
hsa04151	PI3K-Akt signaling pathway
hsa04152	AMPK signaling pathway
hsa04210	Apoptosis
hsa04216	Ferroptosis
hsa04217	Necroptosis
hsa04218	Cellular senescence
hsa04260	Cardiac muscle contraction
hsa04261	Adrenergic signaling in cardiomyocytes
hsa04270	Vascular smooth muscle contraction
hsa04310	Wnt signaling pathway
hsa04330	Notch signaling pathway
hsa04340	Hedgehog signaling pathway
hsa04350	TGF-beta signaling pathway

hsa04360	Axon guidance
hsa04370	VEGF signaling pathway
hsa04371	Apelin signaling pathway
hsa04380	Osteoclast differentiation
hsa04390	Hippo signaling pathway
hsa04510	Focal adhesion
hsa04512	ECM-receptor interaction
hsa04520	Adherens junction
hsa04620	Toll-like receptor signaling pathway
hsa04621	NOD-like receptor signaling pathway
hsa04622	RIG-I-like receptor signaling pathway
hsa04668	TNF signaling pathway
hsa04670	Leukocyte transendothelial migration
hsa04912	GnRH signaling pathway
hsa04917	Prolactin signaling pathway
hsa04934	Cushing syndrome
hsa05165	Human papillomavirus infection
hsa05205	Proteoglycans in cancer
hsa05418	Fluid shear stress and atherosclerosis

Table S2. Overlapped genes.

Upregulated Genes	Downregulated Genes
TACR1	TRAPPC13
PCGF1	LOC100506699
EGFL8	SLMO2
PPT2	ZNF729
EHBP1L1	ERCC5
CHD7	DMXL1
CCNJ	OR10C1
MAP3K5	CRIPT
MAP2K2	ZNF552
MAP3K1	SLC3A1
PF4V1	ISOC1
TRPM4	BBIP1
MAPK1	REV1
COQ4	CHRDL1
PYGB	ZNF107
FOXO6	BMPR1A
FBXO25	RB1
NRAS	RP3-522P13.2
FOXO1	RPAP2
MYL9	APPL1
IRS2	MAP4K3
RAC3	KIF20A
CTNNB1	RCBTB2
UTS2	THAP10
MAGI3	TAAR2
RAPGEF6	RCBTB1
RAP1B	C1orf56
MAP4K1	DYNC1LI1
PLCG1	TRMT61B
PIK3R3	TVP23B
TLR4	MED7
ARAP3	PTER
USP53	UFM1
OSMR	LOC101928620
NR4A1	POU2AF1
MAP2K1	LOC100506282
MAPK3	
GSK3B	
TSPAN2	
PLCG2	
LILRB3	
PUS7L	
LRFN3	
PRKCG	
TSSC4	
STYK1	
PHF8	

TP53
PRKD1
CDKN1A
E2F1
AIF1
FOXM1
DPP3
SNX17
CD320
